# Supplementing Oregano Essential Oil in a Reduced-Protein Diet Improves Growth Performance and Nutrient Digestibility by Modulating Intestinal Bacteria, Intestinal Morphology, and Antioxidative Capacity of Growing-Finishing Pigs

**DOI:** 10.3390/ani8090159

**Published:** 2018-09-19

**Authors:** Chuanshang Cheng, Mao Xia, Xiaming Zhang, Chao Wang, Siwen Jiang, Jian Peng

**Affiliations:** 1 Department of Animal Nutrition and Feed Science, College of Animal Science and Technology, Huazhong Agricultural University, Wuhan 430070, China; chengcs1989@gmail.com (C.C.); a1159075276@163.com (M.X.); 15827298763@163.com (X.Z.); jijingdexiaogelou@163.com (C.W.); 2 The Cooperative Innovation Center for Sustainable Pig Production, Wuhan 430070, China

**Keywords:** antibiotic alternative, reduced-protein diet, oregano essential oil, intestinal bacteria, antioxidative capacity, pigs

## Abstract

**Simple Summary:**

Serious nitrogen pollution and shortage of protein feed resources have constrained the rapid development of the pig industry. Thus, the strategy of using a reduced-protein diet supplemented with amino acids in pig production has been widely accepted. Additionally, antibiotic growth promoters have been widely used in pig production for many years. However, their enormous and uncontrolled use has promoted bacterial resistance, leading to less effective treatment for animal diseases. Accordingly, this study investigated the effects of inclusion of oregano essential oil in a reduced-protein, amino acid-supplemented diet on growth performance, nutrient digestibility, gut health, and antioxidative capacity of growing-finishing pigs as an alternative to antibiotics. Our results suggested that adding oregano essential oil to a reduced-protein diet improved the growth performance and carcass lean percentage of pigs. In addition, long-term supplementation of oregano essential oil to a reduced-protein diet improved the intestinal bacteria, intestinal morphology, and antioxidative capacity of pigs. This study provides theoretical guidance for application of low-protein diet to guide the production of antibiotic-free feed for growing–finishing pigs.

**Abstract:**

This study investigated the effects of supplementing oregano essential oil (OEO) to a reduced-protein diet on growth performance, nutrient digestibility, intestinal bacteria, intestinal morphology, and antioxidative capacity of growing-finishing pigs. Forty-eight barrows were randomly allotted to four treatments including normal-protein diet (NPD), reduced-protein, amino acid-supplemented diet (RPD), the same RPD supplemented with chlortetracycline (RPA), and RPD supplemented with OEO (RPO). The data showed that dietary OEO supplementation increased the average daily gain of pigs compared with NPD and RPD. The gain:feed in RPO- and NPD-fed pigs was higher than those in RPD- and RPA-fed pigs. Increased average daily feed intake and 10th-rib backfat thickness were detected in RPA-fed pigs. Pigs fed the RPO had higher apparent total tract digestibility (ATTD) of crude protein than those fed the other diets. The RPD and RPA treatments showed reduced counts of *Lactobacillus* spp. in ileal digesta of pigs. The RPA and RPO treatments also showed lower *Escherichia coli* counts in ileal digesta than the NPD and RPD treatments. Dietary OEO supplementation increased villous height of the jejunum and the ileal and plasma total antioxidative capacity of pigs. In conclusion, dietary OEO supplementation could improve the growth performance and nutrient digestibility of pigs by modulating intestinal bacteria, intestinal morphology, and antioxidative capacity.

## 1. Introduction

In China, serious nitrogen pollution and shortage of protein feed resources have severely constrained the rapid development of pig industry [1,2]. To address this issue, the low protein diet balanced with supplemental amino acids (AAs) has been widely used in pig production [3]. Several studies have demonstrated that a reduced-protein, amino acid-supplemented diet may reduce feed costs and nitrogen excretion without any negative impact in the performance of pigs [4,5]. 

In addition to nitrogen pollution and protein resource shortage, the use of antibiotic growth promoters has promoted bacterial resistance, leading to less efficient treatments for animal bacterial diseases [6,7]. Several countries in Europe have completely banned the addition of antibiotics to livestock feed [8]. In China, although the inclusion of antibiotics in feed for growing-finishing pigs is still a common practice, it has increasingly received safety concerns from consumers. Therefore, further researches are needed to develop effective and safe alternatives for growth and disease prevention in pigs. As desirable alternatives to antibiotics, herbal feed additives have been reported to improve performance of pigs by enhancing their intestinal health [9]. Oregano essential oil (OEO) is mainly composed of natural and volatile aromatic compounds, which have antimicrobial and antioxidative activities [10]. Our previous studies suggested that OEO supplementation could reduce the population of *Escherichia coli* in the ileum and improve the small intestinal morphology and antioxidant enzyme activities of pigs [11,12,13]. However, it should be pointed out that the basal diet for these studies was a normal protein diet that met or exceeded the NRC [14] nutrient requirement estimates. Recent studies have shown that dietary protein levels affect pig intestinal bacteria, intestinal morphology, and digestive function [12]. Moreover, the activity of antibiotics and their alternatives (such as probiotics) was reported to be affected by dietary protein supply [15,16]. However, no research is available about the influences of OEO inclusion in a reduced-protein diet on growth performance, gut microbiota, intestinal morphology, and antioxidative capacity of growing-finishing pigs. Moreover, the effect of adding OEO as an alternative to antibiotics to a reduced-protein diet on the performance and health status of pigs also remains largely unknown.

Accordingly, the objective of this study was to evaluate the influences of OEO inclusion in a reduced-protein, amino acid-supplemented diet on growth performance, carcass traits, nutrient digestibility, intestinal bacteria, intestinal morphology, and antioxidative capacity of growing-finishing pigs as an alternative to antibiotics. Our hypothesis was that the supplementation of OEO in a reduced-protein diet may improve the growth performance and nutrient digestibility of growing-finishing pigs by positively modulating intestinal bacteria, intestinal morphology, and antioxidative capacity. 

## 2. Material and Methods

The study protocol was approved by the animal care of Huazhong Agricultural University, and the treatment and slaughtering conditions were in accordance with the Animal Care and Use Guidelines of China (The ethical code: HAZUSW-2016-039). 

### 2.1. Animals, Diets, and Experimental Design

A total of forty-eight growing barrows (Large White × Landrace) with initial body weight (BW) of 29.61 ± 1.02 kg were obtained from a pig farm. All pigs were allocated to one of the four treatment groups based on BW by using a completely random experimental design. Each treatment had twelve replicates with one pig per replicate. The size of individual pen was 2.0 × 1.5 meters. The four treatments consisted of normal protein diet (NPD), reduced-protein, amino acid-supplemented diet (RPD), the same RPD supplemented with 150 mg of chlortetracycline per kg of feed (RPA), and the same RPD supplemented with 250 mg of OEO per kg of feed (RPO). The pigs were fed in two dietary phases as follows: 1) from 30 to 65 kg BW as growing period, and 2) from 66 to 115 kg BW as finishing period. The NPD diet contained 17% and 15.6% crude protein during the growing and finishing stages, respectively. The dietary protein levels in these two stages with one percentage unit lower than the NRC [14] nutrient requirement estimates. The RPD diet contained 15% and 13.6% crude protein during growing and finishing stages, respectively. The Lys, Met, Thr, and Trp of all diets were balanced to meet the requirements of growing-finishing pigs [17]. The compositions of feed ingredient and nutrient contents of the experimental diets were shown in Table 1. The chlortetracycline was provided by Jinhe Biotechnology Co., Ltd. (Hohhot, China). The addition form of OEO is a powder called Phytogen (Meritech Bioengineering Co, Guangzhou, China). The Phytogen contains 90% natural feed grade inert carrier and 10% OEO of Greek Origanum Vulgare subsp. hirtum. The dosage of OEO additives was referenced from our previous studies [13,18]. The detailed chemical components of OEO are presented in our previous study [13]. Throughout the study, all pigs were given free access to water and feed via nipple drinkers and semi-automatic individual feeders, respectively. The temperature of the pig pens was controlled within the range of 18 and 23 °C. 

### 2.2. Growth Performance and Nutrient Digestibility Analysis

After adapting to dietary treatments for 5 days, the pigs were fed with experimental diets for 98 days. One pig from the NPD treatment and one from the RPO treatment were excluded because of sudden death due to acute respiratory disease at the beginning of week 1. The pigs were individually weighed on days 0, 49, and 98. Feed intake was recorded, and ADFI, ADG, and gain:feed (G:F) were calculated per pig. Chromic oxide (0.2%) was supplemented to diets as an indigestible marker for determining the apparent total tract digestibility (ATTD) of dry matter, gross energy, and CP from day 36 to 42 and day 85 to 91. The fecal samples of all pigs were collected for 2 consecutive days, from day 41 to 42 and day 90 to 91, respectively, during the feeding period in the morning by rectal stimulation. All fecal samples and feed were stored at −20 °C until analysis. Before chemical analysis, the fecal samples were thawed and dried for 72 h at 70 °C, then they were finely ground and passed through a 1 mm sieve. Additionally, feed samples were ground and passed through a 1 mm screen. All feed and fecal samples were analyzed for dry matter (Method 934.01) according to the AOAC method [19]. Nitrogen content was detected using the Kjeldahl methodology (Method 954.01) based on the AOAC standard [19] and CP was calculated as N × 6.25. Gross energy was determined using an automatic adiabatic oxygen bomb calorimeter (Moline, IL, USA). Chromium values were measured using an atomic absorption spectrophotometer (Tokyo, Japan). The ATTD was then calculated according to the method of Sauer and Lange [20].

### 2.3. Carcass Measurement

On day 98, six barrows per treatment were transported to a commercial slaughterhouse (Ezhou, China) and slaughtered at the same age (173 d of age) and a live weight of 105.95 ± 2.34 kg. Diets were withheld from the pigs 12 h before slaughter. Pigs were exsanguinated, segmented, and measured according to the standard commercial procedures. After a 24-h postmortem chilling period, standard carcass characteristics including hot carcass weight (HCW), dressing percentage, backfat thickness (the 1st rib, 10th rib, and last rib), loin eye area (LEA), and carcass lean percentage (CLP) were determined. The LEA and CLP were measured according to the procedures described by Huang et al. [21].

### 2.4. Sample Collection

Blood samples were collected from the jugular vein using a 0.9-mm-diameter needle into heparinized tubes (5 mL). Blood samples were then centrifuged at 4 °C at 3000× *g* for 10 min. The plasma samples in supernatant were then collected and stored at −80 °C until analysis. The digesta samples (about 4 g) were immediately removed from the proximal ileum of each pig and stored at −80 °C until further analysis. The jejunum and ileum (3 cm) samples were taken from the middle section of them and then rinsed with ice-cold saline. Jejunum and ileum samples were preserved in a 4% neutral-buffered formalin for intestinal morphological analysis.

### 2.5. Determination of Jejunal and Ileal Morphology

The samples of jejunum and ileum were cut to a thickness of 5 mm and then stained with hematoxylin and eosin. The villous height and crypt depth were determined on the stained sections using an optical microscope equipped with an image analyser (Image Pro Plus 6.0, Bethesda, Rockville, MD, USA). Twenty villi and crypts were measured for each segment. The villus/crypt ratio was calculated by dividing villus height by crypt depth. 

### 2.6. Detection of Plasma Antioxidant Enzyme Activity and Oxidative Status

The activities of total antioxidative capacity (T-AOC), glutathione peroxidase (GPx), total superoxide dismutase (T-SOD), and catalase (CAT) and the plasma concentrations of thiobarbituric acid reactive substances (TBARS) were detected using colorimetric methods with a spectrophotometer (Biomate 5, Rochester, NY, USA). The assays were carried out using commercial kits (Nanjing Jiancheng Bioengineering Institute, Nanjing, Jiangsu, China) and their corresponding procedures. The assays were performed in triplicate. 

### 2.7. Quantification of Selected Ileal Bacteria

Total microbial DNA was extracted and purified from the ileal digesta using a QIAamp DNA stool kit (Qiagen, Hilden, Germany) according to the manufacturer’s instructions. Genomic DNA from ileal digesta was collected and amplified by conventional PCR amplification using species and genus specific primers (Table S1). Primers used in this study were either synthesized according to our previous protocols or designed with Primer 5.0 according to pig gene sequences. After PCR amplification, the products of PCR were purified according to the manufacturer’s method (Omega, Norcross, GA, USA). The purified PCR products were linked to the pMD 18-T vector system (Takara, Japan) and then transferred to Escherichia coli DH5α (Qiagen, Hilden, Germany) for cloning. The positive clone plasmids were commercially sequenced to obtain the positive plasmids (Shanghai Sangon Biological Engineering Technology Service Co. Ltd., Shanghai, China). The plasmids were extracted and purified using the Qiagen Plasmid Midi kit (Qiagen, Hilden, Germany) and quantified by a spectrophotometer (Thermo Co., Vantaa, Finland). A series of dilutions of these positive plasmids were used to generate standard curves using quantitative real-time PCR (Bio-Rad, Berkeley, CA, USA), allowing absolute quantification to be estimated based on the respective gene copies. Absolute quantitative real-time PCR was carried out referring to the method of Wei et al. [22]. The data are presented as gene copy numbers per gram of wet faeces and shown as Log10 cfu/g intestinal content for data analysis.

### 2.8. Statistical Analysis

The data were analysed using the general linear model (GLM) procedure of the Statistical analysis system v. 8.1 (SAS Inst., Inc., Cary, NC, USA). Each pig was considered as an experimental unit. An ANOVA model, with the dietary treatment (NPD, RPD, RPA, and RPO) as fixed factor, was used for all data. Differences among means were detected by the method of Duncan’s Multiple Range Test [23]. The means were calculated using the least square method and expressed as means ± standard errors of the means (SEM). Significance for all analyses is indicated at a *p* ≤ 0.05.

## 3. Results

### 3.1. Growth Performance

The data of growth performance in growing-finishing pigs are shown in Table 2. During growing period, pigs fed with the RPA exhibited the highest ADFI and the lowest (*p* < 0.01) G:F. Pigs fed with NPD exhibited higher (*p* < 0.01) G:F than those receiving RPD but similar to those fed with the RPO. During finishing stage, pigs receiving RPO diet exhibited higher final BW (*p* < 0.05) and ADG (*p* = 0.055) than those receiving NPD and RPD but similar values to those fed with the RPA. RPA-fed pigs showed greater (*p* < 0.05) final BW than the NPD-fed pigs. Pigs fed with the RPA had greater (*p* < 0.05) ADFI than those fed with NPD. RPO- and NPD-fed pigs had higher (*p* < 0.01) G:F than pigs receiving RPD and RPA. Overall, pigs receiving the RPO exhibited greater (*p* < 0.05) ADG and lower (*p* < 0.01) adjusted days to 110 kg of BW than those fed with NPD and RPD pigs but similar values to those fed with RPA. Pigs fed with RPA had the greatest (*p* < 0.01) ADFI. RPO- and NPD-fed pigs had higher (*p* < 0.05) G:F than pigs receiving RPD and RPA. 

### 3.2. Apparent Total Tract Digestibility

The data of apparent total tract digestibility data are presented in Table 3. During growing and finishing period, pigs fed with RPO and NPD had higher (*p* < 0.05) ATTD of dry matter than pigs fed with RPD and RPA. Additionally, pigs fed with RPO had the highest (*p* < 0.01) ATTD of CP while pigs fed with RPA had the lowest ATTD of CP. The ATTD of CP was not different (*p* > 0.05) between NPD and RPD treatments. Additionally, the ATTD of gross energy was not different among four treatments (*p* > 0.05).

### 3.3. Carcass Characteristics

The effects of dietary treatments on carcass characteristics are shown in Table 4. The RPA treatment exhibited the greatest (*p* < 0.05) backfat thickness in 10th rib compared with other three treatments. Additionally, The CLP was greater (*p* < 0.05) in pigs fed with RPO than those receiving NPD and RPA. No different was shown on HCW, dressing percentage, Backfat thickness (1st rib and last rib), and LEA among different dietary treatments.

### 3.4. Selected Microbial Populations in Ileal Digesta

**Figure 1:**
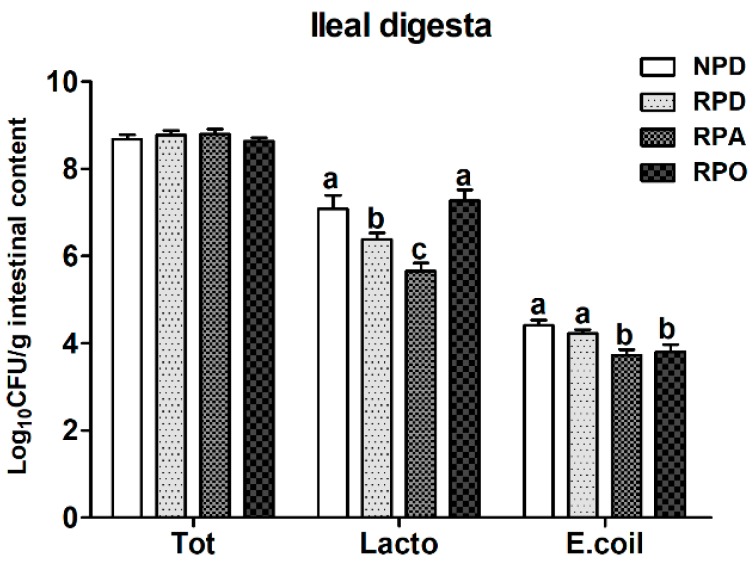
Influences of inclusion of oregano essential oil in a low-protein diet on intestinal microbiota of growing-finishing pigs. ^a,b^ Letters within a row indicate dramatical differences among means (*p* < 0.05). ^1^ Values represent the mean of 6 pigs during the trial. ^2^ NPD = normal protein group; RPD = reduced protein, amino acid-supplemented group; RPA = 150 mg/kg chlortetracycline group; RPO = oregano essential oil (250 mg/kg) group; *E. coli* = *Escherichia coli.*

The influences of inclusion of oregano essential oil in a reduced-protein diet on selected microbial populations in ileal digesta are shown in Figure 1. Dietary treatments had no significant effect on total bacteria. The NPD- and RPO-fed pigs had a higher (*p* < 0.05) population of *Lactobacillus* in ileal digesta than those in the RPD- and RPA-fed pigs. The RPA-fed pigs exhibited the lowest (*p* < 0.05) population of *Lactobacillus*. In addition, the population of *Escherichia coli* in the RPA and RPO treatment was lower than in the NPD and RPD treatment.

### 3.5. Morphology of the Jejunum and Ileum

The histomorphometrical intestinal measurement is summarized in Table 5. There was no significant difference in crypt depth and the ratio of villus height to crypt depth in the jejunum and ileum between different treatments (*p* > 0.05). The villous height of the jejunum and ileum in RPO-fed pigs was significantly higher than that of other three treatments (*p* < 0.01).

### 3.6. Antioxidant Activity and Lipid Peroxidation in Plasma

Table 6 shows the influences of supplementing oregano essential oil to a reduced-protein diet on plasma TBARS concentrations and plasma antioxidant enzymes activity. In plasma, the levels of TBARS and CAT were unaffected (*p* > 0.05) by dietary treatments. Pigs fed with RPO exhibited the highest activities of T-AOC, T-SOD, and GPx than pigs fed with the NPD, RPD, and RPA (*p* < 0.05).

## 4. Discussion

Severe nitrogen pollution and the use of antibiotic growth promoters have adversely affected the rapid development of the pig industry and human health [1,6]. Therefore, developing antibiotic alternatives on the basis of a reduced-protein, amino acid-supplemented diet may be one of the effective measures to solve these problems. In this current study, our results suggest that although growth rates of growing-finishing pigs were maintained, the feed efficiency of pigs was significantly reduced when the dietary protein concentration was reduced by 2% units while balancing the first four limiting AAs to meet the NRC [17] requirements. Several studies have demonstrated that the reduction of the CP concentration by 2% units with crystalline AA inclusion did not reduce the feed conversion rate in pigs [12,24]. One of the possible reasons for the inconsistency is that the energy systems used to formulate diets are different. For example, the energy systems used by Zhou et al. [12] and Madrid et al. [24] were digestive energy and metabolizable energy, respectively. However, in this study, the net energy (NE) system was used. Noblet et al. [25] demonstrated the negative effect of protein content on the net energy value of diets. Moreover, Kerr et al. [26] suggested that dietary CP concentration interacted with NE for gain:feed of pigs and long-term feeding of low-protein diets on a low NE basis reduced feed conversion rate in growing-finishing pigs. More importantly, the reduction in crude protein levels from NRC [14] to RPD was about 3%, but only four major essential amino acids were at the same levels in RPD as in NPD. In this case, the supply of other “nutritionally nonessential AA” is insufficient [27]. These results further emphasize the important effect of synthetic AA in maintaining growth and development of pigs. Additionally, RPD-fed pigs had lower ATTD of dry matter than NPD-fed pigs. This result agreed with the finding of a study [27] that reported that the expression of genes encoding digestive enzymes (such as trypsinogen and dipeptidases-II and III) in the intestine is reduced after feeding a low-protein diet. Interestingly, the RPD-fed pigs had a lower population of *Lactobacillus* in ileal digesta than the NPD-fed pigs. Similarly, Zhou et al. [12] suggested that a low protein diet significantly reduced the abundances of *Lactobacillus* in gut of pigs. *Lactobacillus*, a member of the lactic acid bacteria group, is considered to improve the health of intestine, thus protecting the intestine from pathogens and promoting the effective extraction of nutrient and energy in animal’s body [28]. It has been showed that *Lactobacillus* was enriched in finishing pigs with high feed efficiency [29]. Thus, we suggest that RPD treatment reduces population of *Lactobacillus* in the ileum, thereby affecting the nutrient digestibility of dry matter and feed conversion of pigs. Considering the importance of *Lactobacillus* in the health of pigs [30], further studies are needed to evaluate the role of a long-term reduced-protein, amino acid-supplemented diet in intestinal microbiota of growing-finishing pigs.

Furthermore, our results indicated that pigs fed with RPA exhibited the highest ADFI and the backfat thickness in 10th rib but the lowest ATTD of CP of pigs. In normal diets, chlortetracycline has been widely added to feed as the growth promoter to increase growth rate and feed conversion of pigs [31]. Recent studies have shown that the activity of antibiotics was affected by dietary protein supply [15,16]. To the best of our knowledge, there is little data available investigating the effects of chlortetracycline supplementation in reduced-protein, amino acid-supplemented diets on growth performance, nutrient digestibility, and carcass traits of growing-finishing pigs. In the current study, with similar ATTD of gross energy, RPA treatment significantly increases feed intake of pigs, which in turn causes excess energy to eventually lead to deposition of fat at the 10th rib. Similarly, Cho et al. [32] have pointed that exposure of mice to subtherapeutic doses of antibiotics also increased total and relative body fat mass. However, further research is needed to clarify the underlying mechanism of RPA leading to increased feed intake. Our data also suggest that subtherapeutic doses of chlortetracycline supplementation in reduced-protein diet may have no significant effect on improving growth performance of growing-finishing pigs. Additionally, the RPA-fed pigs had a lower population of *Lactobacillus* and *Escherichia coli* in ileal digesta compared with the RPD-fed pigs. These results are consistent with those of Allen et al. [33] and Li et al. [34], who reported that that population of *E. coli* in intestine is decreased in chlortetracycline-treated pigs and broilers, respectively. These results suggest that RPA treatment broadly reduces the population of *Escherichia coli* and probiotic bacteria. 

In this study, low-protein diet supplemented with OEO improved the growth rate and feed conversion and shortened the slaughter day of fattening pigs. Several previous studies reported the positive role of OEO on the growth performance of growing-finishing pigs on the basis of normal protein diet [18,35,36]. However, no research was published concerning the effects of inclusion of OEO in low-protein diet on growth performance of pigs. Oregano essential oil (OEO), mainly composed of natural and volatile aromatic compounds, can exert antimicrobial and antioxidative activities [10]. Indeed, the improved digestive function in RPO treatment was confirmed by higher ATTD of dry matter and CP during growing and finishing period in this study. Our results agree with the literatures reporting the facilitating roles of OEO on digestive function in pigs [37]. Additionally, the improvement in nutrient absorption of OEO may be partly explained by increased secretions of saliva and bile and enhanced enzyme activities [38,39]. Interestingly, we found that RPO diet increased the carcass lean percentage of growing-finishing pigs, which consistent with the result of other researcher [40] who showed that OEO diet increased breast muscle percentage in chickens. To some extent, RPO may promote the deposition of protein in muscle by improving CP digestion and absorption, thereby promoting lean growth. Further prospective studies are needed to elucidate the underlying mechanisms by which RPO promotes lean growth. In addition, dietary RPO improved the villous height of jejunum and ileal, circulating antioxidant enzymes (such as T-SOD, T-AOC, and GPx), and the populations of *Lactobacillus* of ileal digesta and reduced plasma TBARS levels in pigs. TBARS is an end product of lipid peroxidation [41]. The reduced plasma TBARS levels indicated that RPO could depress the degree of lipid peroxidation in growing-finishing pigs. Moreover, the increased activities of circulating antioxidant enzymes suggested that RPO can protect pigs against oxidative stress by improving their antioxidant defence system. A lower oxidative stress status will be more conducive to the growth of fattening pigs. An increase in the height of villi can be regarded as indicator of improved nutrient absorption capacity [42]. No previous research was published concerning the intestinal morphology and gut microbiota of pigs fed with low-protein diet supplemented with OEO. Our previous studies indicated that OEO can modulate intestinal bacteria and improve the small intestinal morphology and antioxidant enzymes activities of pigs on basis of normal protein diet [11,12,13]. These results confirm the hypothesis that the use of OEO as an alternative to antibiotics in reduced-protein diets can improve intestinal epithelial absorptive function, gut microbiota composition, and antioxidative stress capacity in growing-finishing pigs. This study provides theoretical guidance for application of low protein diet technology to guide the production of antibiotic-free feed.

## 5. Conclusions

In the present study, our results suggest that supplementing oregano essential oil to a reduced-protein, amino acid-supplemented diet effectively improve the growth performance, nutrient digestibility, and carcass lean percentage of growing-finishing pigs. The long-term adding oregano essential oil to a reduced-protein, amino acid-supplemented diet has the potential to modulate intestinal bacteria and improve intestinal morphology and antioxidative capacity of growing-finishing pig. Besides, this study provides theoretical guidance for application of low protein diet technology to guide the production of antibiotic-free feed.

## Figures and Tables

**Table 1:** The ingredient compositions and nutrient analysis of the experimental diets (%, as-fed basis) ^1^.

Diets (%)	Growing Stage		Finishing Stage	
	NPD	RPD	NPD	RPD
Corn	60.55	65.35	65.72	69.81
Soybean meal	16.20	9.70	13.72	7.20
DDGS ^2^	12.50	12.50	10.00	10.00
Wheat bran	6.00	7.30	6.77	8.70
Soybean oil	1.21	1.21	0.80	0.80
CaHPO_4_	0.70	0.80	0.60	0.60
Limestone	0.80	0.80	0.60	0.80
Salt	0.36	0.36	0.36	0.36
L-Lysine·HCl	0.49	0.67	0.35	0.53
DL-Met	0.04	0.06	0.00	0.01
L-Thr	0.11	0.18	0.06	0.15
L-Trp	0.04	0.07	0.02	0.04
1% Premix ^3^	1.00	1.00	1.00	1.00
Calculated nutrients				
Net energy ^4^, Kcal/kg	2475	2475	2475	2475
Crude protein, %	17.00	15.00	15.60	13.60
Standardized ileal digestible amino acids ^5^, %				
Lys	0.98	0.97	0.81	0.82
Met	0.28	0.28	0.22	0.21
Met + Cys	0.55	0.53	0.47	0.45
Thr	0.59	0.58	0.50	0.51
Trp	0.17	0.17	0.14	0.13
Analysed nutrients, %				
Crude protein	17.02	15.03	15.62	13.60
Dry matter	87.20	87.40	88.13	88.45
Crude fiber	3.85	3.78	3.62	3.56
Total ash	6.10	6.13	5.80	5.94
Ether extract	4.10	4.09	4.15	4.13
Ca	0.60	0.59	0.58	0.57
Total P	0.42	0.42	0.40	0.41

^1^ NPD, normal protein diet; RPD, reduced-protein diet. ^2^ Distillers dried grains with solubles. ^3^ Premix provided these amounts of vitamins and minerals per kilogram on an as-fed basis: vitamin A, 11,500 IU; vitamin D3, 3500 IU; vitamin E, 35 IU; vitamin K3, 5 mg; vitamin B1, 7 mg; vitamin B2, 10 mg; vitamin B6, 8 mg; vitamin B12, 0.06 mg; biotin, 0.16 mg; folic acid, 2 mg; niacin, 50 mg; D-calcium pantothenate, 30 mg; Fe, 150 mg as ferrous sulfate; Cu, 130 mg as copper sulfate; Mn, 40 mg as manganese oxide; Zn, 110 mg as zinc oxide; I, 0.6 mg as potassium iodide; and Se, 0.4 mg as sodium selenite. ^4,5^ According to National Research Council (2012).

**Table 2:** Effects of supplementing oregano essential oil to a reduced-protein diet on growth performance of growing-finishing pigs ^1^.

Item ^2^	NPD	RPD	RPA	RPO	SEM	*p*-Value
***Growing Period***						
Initial BW, kg	29.69	29.67	29.56	29.52	0.15	0.97
Final BW, kg	62.52	62.85	65.48	64.08	0.54	0.20
ADG, kg/d	0.672	0.675	0.733	0.707	0.01	0.13
ADFI, kg/d	1.66 ^b^	1.79 ^b^	2.14 ^a^	1.80 ^b^	0.04	<0.01
G:F, kg/kg	0.408 ^a^	0.377 ^b^	0.345 ^c^	0.395 ^a,b^	0.01	<0.01
***Finishing Period***						
Final BW, kg	104.52 ^c^	105.72 ^b,c^	111.01 ^a,b^	113.53 ^a^	1.16	0.01
ADG, kg/d	0.857 ^b^	0.865 ^b^	0.929 ^a,b^	1.000 ^a^	0.02	0.055
ADFI, kg/d	2.67 ^b^	2.94 ^a,b^	3.22 ^a^	2.94 ^a,b^	0.07	0.03
G:F, kg/kg	0.322 ^a^	0.293 ^b^	0.289 ^b^	0.343 ^a^	0.01	<0.01
***Overall***						
ADG, kg/d	0.765 ^c^	0.775 ^b,c^	0.831 ^a,b^	0.857 ^a^	0.01	0.01
ADFI, kg/d	2.17 ^b^	2.38 ^b^	2.63 ^a^	2.37 ^b^	0.05	<0.01
G:F, kg/kg	0.355 ^a^	0.327 ^b^	0.316 ^b^	0.363 ^a^	0.00	<0.01
DAYS ^3^	185 ^a^	181 ^a,b^	174 ^b,c^	170 ^c^	1.88	0.02

^a,b^ Means in the same row with the same or no superscript letter do not differ, *p* > 0.05. ^1^ Values represent the mean of 11–12 pigs during the trial. ^2^ NPD = normal protein group; RPD = reduced protein, amino acid-supplemented group; RPA = 150 mg/kg chlortetracycline group; RPO = oregano essential oil (250 mg/kg) group. ^3^ DAYS = adjusted days to 110.0 kg (NSIF, 1997).

**Table 3:** Effects of inclusion of oregano essential oil in a low-protein diet on apparent total tract nutrient digestibility of growing-finishing pigs ^1^.

Item ^2^	NPD	RPD	RPA	RPO	SEM	*p*-Value
***Growing Period***						
Dry matter, %	87.61 ^a^	85.36 ^b^	84.89 ^b^	87.56 ^a^	0.70	0.02
Crude protein, %	82.43 ^b^	82.55 ^b^	79.50 ^c^	84.35 ^a^	0.68	<0.01
Gross energy, %	85.96	85.40	85.33	85.30	1.90	0.78
***Finishing Period***						
Dry matter, %	83.24 ^a^	81.40 ^b^	81.36 ^b^	83.55 ^a^	0.85	0.03
Crude protein, %	79.87 ^b^	80.13 ^b^	76.30 ^c^	82.10 ^a^	0.65	<0.01
Gross energy, %	81.05	81.15	81.23	81.43	0.10	0.80

^a,b^ Means in the same row with different letters differ dramatically (*p* < 0.05). ^1^ Values represent the mean of 11–12 pigs during the trial. ^2^ NPD = normal protein group; RPD = reduced protein, amino acid-supplemented group; RPA = 150 mg/kg chlortetracycline group; RPO = oregano essential oil (250 mg/kg) group.

**Table 4:** Influences of inclusion of oregano essential oil in a low-protein diet on carcass traits of growing-finishing pigs ^1^.

Item ^2^	NPD	RPD	RPA	RPO	SEM	*p*-Value
Slaughter body weight, kg	104.92	106.22	107.50	105.17	0.48	0.21
HCW, kg	79.14	79.65	79.87	78.18	0.43	0.55
Dressing percentage	75.52	74.98	74.28	74.35	0.25	0.29
Backfat thickness, mm						
1st rib	34.37	35.16	34.57	35.82	0.92	0.96
10th rib	16.55 ^b^	17.55 ^b^	23.00 ^a^	15.06 ^b^	0.86	<0.01
last rib	13.25	12.90	15.92	13.96	0.71	0.46
LEA, cm^2^	45.20	48.26	47.53	48.27	0.68	0.33
CLP, %	61.75 ^b^	64.03 ^a,b^	61.53 ^b^	65.82 ^a^	0.62	0.04

^a,b^ Means in the same row with different letters differ dramatically (*p* < 0.05). ^1^ Values represent the mean of 6 pigs during the trial. ^2^ NPD = normal protein group; RPD = reduced protein, amino acid-supplemented group; RPA = 150 mg/kg chlortetracycline group; RPO = oregano essential oil (250 mg/kg) group; HCW = hot carcass weight; LEA = loin eye area; CLP = carcass lean percentage.

**Table 5:** Influences of inclusion of oregano essential oil in a low-protein diet on intestinal morphology of growing-finishing pigs ^1^.

Item ^2^	NPD	RPD	RPA	RPO	SEM	*p*-Value
**Jejunum**						
Villous height, um	372 ^b^	375 ^b^	392 ^b^	432 ^a^	6.71	<0.01
Crypt depth, um	212	211	217	222	4.40	0.84
Villous height: crypt depth	178	1.81	1.82	1.96	0.05	0.62
**Ileum**						
Villous height, um	327 ^b^	339 ^b^	352 ^b^	388 ^a^	6.54	<0.01
Crypt depth, um	182	183	186	197	4.65	0.64
Villous height: crypt depth	1.85	1.88	1.91	1.97	0.05	0.89

^a,b^ Letters within a row indicate dramatical differences among means (*p* < 0.05). ^1^ Values represent the mean of 6 pigs during the trial. ^2^ NPD = normal protein group; RPD = reduced protein, amino acid-supplemented group; RPA = 150 mg/kg chlortetracycline group; RPO = oregano essential oil (250 mg/kg) group.

**Table 6:** Influences of inclusion of oregano essential oil in a low-protein diet on antioxidative enzyme activities in plasma of pigs ^1^.

Item ^2^	NPD	RPD	RPA	RPO	SEM	*p*-Value
TBARS, nmol/mL	4.67	4.58	4.46	4.40	0.07	0.69
T-AOC, U/mL	3.49 ^b^	3.59 ^b^	3.55 ^b^	4.32 ^a^	0.10	<0.01
T-SOD, U/mL	129 ^b^	139 ^b^	133 ^b^	159 ^a^	3.62	<0.01
CAT, U/mL	571	577	563	582	4.93	0.56
GPx, U/mL	616 ^b^	641 ^b^	625 ^b^	722 ^a^	11.22	<0.01

^a,b^ Letters within a row indicate dramatical differences among means (*p* < 0.05). ^1^ Values represent the mean of 6 pigs during the trial. ^2^ NPD = normal protein group; RPD = reduced protein, amino acid-supplemented group; RPA = 150 mg/kg chlortetracycline group; RPO = oregano essential oil (250 mg/kg) group; MDA = malondialdehyde; T-SOD = total superoxide dismutase; T-AOC = total antioxidative capacity; GPx = glutathione peroxidase; CAT = catalase.

## Supplementary Materials

The following are available online at http://www.mdpi.com/2076-2615/8/9/159/s1, Table S1: Absolute quantitative real-time PCR primers for detecting microbial populations in faeces of finishing pigs.
