# Supplementary material for: Supplementing Oregano Essential Oil in a Reduced-Protein Diet Improves Growth Performance and Nutrient Digestibility by Modulating Intestinal Bacteria, Intestinal Morphology, and Antioxidative Capacity of Growing-Finishing Pigs
Source: Animals (Basel). 2018 Sep 19;8(9):159. doi: 10.3390/ani8090159 (PMC6162377; doi:10.3390/ani8090159)
Supplement: Supplementary file 1 [file animals-08-00159-s001.docx]

**Table S1.** Absolute quantitative real-time PCR primers for detecting microbial populations in faeces of finishing pigs.

| Target group | Sequence of primers (5^′^ to 3^′^) | Size (bp) | Annealing temperature (℃) |
| --- | --- | --- | --- |
| Total bacteria | Forward: ACTCCTACGGGAGGCAGCAG  Reverse: ATTACCGCGGCTGCTGG | 175 | 60 |
| *Lactobacillus spp.* | Forward: CACCGCTACACATGGAG | 341 | 58 |
|  | Reverse: TGGAAGATTCCCTACTGCT |  |  |
| *Escherichia coli* | Forward: CATGCCGCGTGTATGAAGAA | 96 | 60 |
|  | Reverse: TTTGCTCATTGACGTTACCCG |  |  |
